# Supplementary material for: Spatio-Temporally Efficient Coding Assigns Functions to Hierarchical Structures of the Visual System
Source: Front Comput Neurosci. 2022 May 27;16:890447. doi: 10.3389/fncom.2022.890447 (PMC9184804; doi:10.3389/fncom.2022.890447)
Supplement: Supplementary file 1 [file Data_Sheet_1.docx]

Supplementary Material for “Spatio-temporally efficient coding assigns functions to hierarchical structures of the visual system”

# Summary for all mathematical formulations

$X$ : neural responses.

$S$ : stimulus.

$H\left( X \right)$ : the entropy of $X$.

$H\left( X|S \right)$ : the conditional entropy of $X$ given $S$.

$I\left( X;S \right)=H\left( X \right)-H\left( X|S \right)$ : the Shannon mutual information between $X$ and $S$.

$X_{h,t}$ : neural responses of the hierarchy $h$ at time $t$, vector form.

$X_{image,t}$ or $X_{h=0,t}$ : an external input image at time $t$, vector form.

$f_{t}$ : a neural responses function from time $t-1$ to time $t$ on the hierarchy, which indicates the inference.

$$\begin{matrix} f_{t}:X_{image, t-1}\times X_{h=1, t-1}\times\ldots\times X_{h=H, t-1}\to X_{image, t}\times X_{h=1, t}\times\ldots\times X_{h=H, t} & (1) \end{matrix}$$

$f_{t}\left( \cdot\right)\left. \right|_{X_{h,t}}$ : the restriction of the range of the function value $f_{t}\left( \cdot\right)$ to $X_{h,t}$.

$W_{h,h'}$ : a synaptic weight matrix from hierarchy $h$ to $h'$, matrix form.

$b_{h}$ : a bias vector at hierarchy $h$.

$\sigma\left( \cdot\right)$ : the sigmoid function.

If $h>0$,

$$\begin{matrix} f_{t}\left. \right|_{X_{h,t}}=\sigma\left( W_{h+1,h}^{T}X_{h+1,t-1}+W_{h,h}^{T}X_{h,t-1}+W_{h-1,h}^{T}X_{h-1,t-1}+b_{h} \right). & (2) \end{matrix}$$

If $h=0$,

$$\begin{matrix} f_{t}\left. \right|_{X_{h,t}}=\sigma\left( W_{h+1,h}^{T}X_{h+1,t-1}+b_{h} \right). & (3) \end{matrix}$$

$L_{Temporal}$ : the objective function of temporally efficient coding, which is the minimisation of temporal differences of neural responses.

$f_{t+1}\circ f_{t}$ : the composition of two functions: $f_{t}$ and $f_{t+1}$.

$x_{h=0,\tilde{t},\mathrm{image}=n}$ : indication that the input image is fixed to the $n$th sample throughout the temporal processing.

$$\begin{matrix} L_{Temporal}=\sum_{n=1}^{N} \left( f_{t}\circ f_{t-1}\left( x_{h=0,\tilde{t},\mathrm{image}=n} \right)-f_{t+1}\circ f_{t}\circ f_{t-1}\left( x_{h=0,\tilde{t},\mathrm{image}=n} \right) \right)^{2} & (4) \end{matrix}$$

$L_{Spatial}$ : the objective function of spatially efficient coding, which is the minimisation of the negative informational entropy.

$P\left( \cdot\right)$ : a probability

$$\begin{matrix} L_{Spatial}=\sum_{n=1}^{N} \log P\left( f_{t}\left( x_{h=0,\tilde{t},\mathrm{image}=n} \right)\left. \right|_{X_{h}} \right) & (5) \end{matrix}$$

$Q\left( x \right)$ : the probabilistic density of neural responses.

$Q'\left( x \right)$ : the probabilistic compensation density of pseudo-uniformly generated samples.

$$\begin{matrix} P\left( x \right)=\frac{Q\left( x \right)}{Q'\left( x \right)} & (6) \end{matrix}$$

The objective function of spatio-temporally efficient coding is

$$\begin{matrix} L=L_{Temporal}+\lambda L_{Spatial} & (7) \end{matrix}$$

where $\lambda$ is the regularization parameter.

$D_{\mathrm{true}}$ : a binary-valued random variable for the discrimination between two actually different inputs such that $D_{\mathrm{true}}=1$ means the discrimination that two noised inputs differ and $D_{\mathrm{true}}=0$ means the discrimination that two noised inputs are same.

$D_{\mathrm{response}}$ : a binary-valued random variable for the discrimination between two neural responses for inputs such that $D_{\mathrm{response}}=1$ means the discrimination that two neural responses differ and $D_{\mathrm{response}}=0$ means the discrimination that two neural responses are same.

$P_{\mathrm{true}}$ or $P_{\mathrm{response}}$ : the probability mass functions of $D_{\mathrm{true}}$ or $D_{\mathrm{response}}$.

$D_{\mathrm{KL}}\left( P_{\mathrm{true}}||P_{\mathrm{response}} \right)$ : the Kullback–Leibler divergence from $P_{\mathrm{response}}$ to $P_{\mathrm{true}}$.

Discriminability for an image $s_{ref}$ = $\left| \left\{ s\in\mathrm{images}| d_{S}\left( s,s_{ref} \right)>\theta_{S} \mathrm{and} d_{X}\left( f\left( s \right),f\left( s_{ref} \right) \right)>\theta_{X} \right\} \right|/\left| \left\{ s\in\mathrm{images}| d_{S}\left( s,s_{ref} \right)>\theta_{S} \right\} \right|$ where $\left| \left\{ \cdot\right\} \right|$ is the set size, $d_{S}\left( s,s_{ref} \right)$ is the distance on the natural scene image space, $d_{X}\left( f\left( s \right),f\left( s_{ref} \right) \right)$ is the distance on the neural response space, $\theta_{S}$ is the threshold which indicates 99% of all images, and $\theta_{X}$ is the threshold which indicates 99% of all neural responses.

$Neuronal noise=\frac{1}{\left| S \right|}\sum_{s\in S} H\left( f_{t}|s,t\in\left[ t^{'}+1,t^{'}+5 \right] \right)$ for each $t^{'}\in\left\{ 0, 5, 10, 15, 20 \right\}$ where $s$ is an image, $S$ is the set of all images, $H\left( \cdot|\cdot\right)$ is the conditional entropy, and $f_{t}$ is the neural response at time $t$.

$Confusion index for image s at time t=\left| f_{t}\left( s \right)-f_{10}\left( s \right) \right|/\left| f_{10}\left( s \right)-f_{10}\left( s' \right) \right|$where $f_{t}\left( s \right)$ is the neural response for image $s$ at time $t$ and $s'$ is the nearest image of $s$ based on the global feature based distance.
